# Pipeline Embolization Device With Adjunctive Coils for the Treatment of Unruptured Large or Giant Vertebrobasilar Aneurysms: A Single-Center Experience

**DOI:** 10.3389/fneur.2020.522583

**Published:** 2020-10-30

**Authors:** Yangyang Zhou, Xinzhi Wu, Zhongbin Tian, Xinjian Yang, Shiqing Mu

**Affiliations:** Department of Interventional Neuroradiology, Beijing Neurosurgical Institute, Beijing Tiantan Hospital, Capital Medical University, Beijing, China

**Keywords:** pipeline embolization device, coils, large aneurysms, giant aneurysms, vertebrobasilar artery

## Abstract

**Objective:** To evaluate effectiveness and safety of Pipeline embolization device (PED) for large or giant verterbrobasilar aneurysms (LGVBAs), and to compare the therapeutic effects of PED with and without adjunctive coils.

**Methods:** We retrospectively analyzed 21 cases of unruptured LGVBAs who were treated in our hospital with PED. These cases were divided into “PED group” and “PED with adjunctive coils group.” We compared the aneurysm characteristics and treatment outcomes between the two groups.

**Results:** The overall neurological complication rate was 28.6% (6/21) and the mortality rate was 4.8% (1/21). There were 12 patients in the PED group and nine in the PED with adjunctive coils group. There were no significant differences in age, smoking, hypertension, aneurysm size, aneurysm location, or operation time between the two groups. The complete aneurysm embolization rate and favorable outcome rate (modified Rankin Scale = 0,1) of the PED with adjunctive coils group was 78% (7/9) and 100% (9/9), respectively, which were both better compared with the PED group with 63.6% (7/11) and 83% (10/12), respectively. However, these differences were not statistically significant.

**Conclusion:** The effectiveness and safety of PED for LGVBAs is acceptable. Treatment results did not differ between the PED and PED with adjunctive coils groups; therefore, whether coils should be used may depend the operator. Our results suggest that correct use of the coils does not increase complications. We suggest that PED with adjunctive coils should be used for some selected LGVBAs.

## Introduction

Since gaining U.S. Food & Drug Administration approval in April 2011 to treat large or giant wide-necked intracranial aneurysms in the internal carotid artery from the petrous to the superior hypophyseal segments, the Pipeline embolization device (PED; Medtronic, Minneapolis, MN, USA) has been wildly used to treat various intracranial refractory aneurysms. Clinical experience has demonstrated the safety and efficiency of PED for internal carotid artery aneurysm, and it has great advantages over traditional embolization methods, such as stent-assisted coil embolization (1–4). PED has also been used to treat posterior vertebrobasilar aneurysms and has achieved acceptable outcomes (5, 6). However, among intracranial aneurysms, posterior vertebrobasilar aneurysms remain the most formidable lesions to treat and clinical outcomes can be poor with a high risk of mortality and disability. This is particularly true for large or giant vertebrobasilar fusiform aneurysms, because some of them progress rapidly and are prone to rupture or can compress the brain stem, even in PED-treated cases (7–9). Moreover, implantation of PED may lead to occlusion of the important vertebrobasilar branch artery because a PED has a 30–35% metal surface area coverage, which can lead to infarction (10, 11). Therefore, there are still no firmly accepted therapeutic methods for using PED. Previous studies (12) have reported that PED combined with coils may be appropriate for some larger aneurysms, and this treatment strategy has worked in the anterior circulation. However, because of the rarity of large or giant vertebrobasilar aneurysm (LGVBA) cases, there is still little experience of PED treatment for LGVBAs. Furthermore, the combined use of coils is also controversial because it can increase the mass effect, which can accelerate aneurysm rupture or compress the brain stem with fatal consequences (13, 14). Therefore, this study attempted to evaluate the effectiveness and safety of PED for LGVBAs, and to compare the therapeutic effects of a single-center PED group with a PED with adjunctive coils group.

## Materials and Methods

### Data Collection and Follow-Up

We retrospectively analyzed 21 cases of LGVBA treated in our hospital with PED between January 2016 and March 2019. The study was approved by the ethics board of Beijing Tiantan Hospital and all patients agreed to participate and signed informed consent forms. All cases had unruptured aneurysms. The cases were divided into two groups based on whether coils were used: a PED group of nine patients and a PED with adjunctive coils group of 12 patients. We defined large aneurysms to have a maximum diameter (saccular aneurysms) or longest diameter (fusiform aneurysms) of between 15 and 25 mm and giant aneurysms to have a maximum or longest diameter >25 mm. We retrospectively analyzed these patients' medical records to obtain major demographic information, including gender, age, and chief complaint. At the same time, we reviewed imaging examinations and the operation process to gain anatomical information of aneurysms: size, location and whether there were extra aneurysms, the number of PEDs used and whether coils were also used.

Post-operative follow-up imaging included digital subtraction angiography (DSA), computed tomographic angiography (CTA), and magnetic resonance angiography (MRA). Generally, patients should undergo the first post-operative examination by DSA within 3–6 months. If the imaging results showed that the aneurysm was completely occluded and the patient displayed no nervous system symptoms, the next examination was by CTA after a longer period of time. Classification of aneurysm embolization was according to O'Kelly–Marotta grading scale (D: complete occlusion; C: trace filling; B: entry remnant; A: aneurysm filling). The modified Rankin scale (mRS) was used to assess clinical outcomes. We went through each patient's medical history and conducted telephone interviews to acquire mRS score. mRS of 0–1 indicated a favorable outcome. Whereas recovery was poor when the score was 4–6.

### Endovascular Procedure

All patients received aspirin (100 mg) and clopidogrel (75 mg) for 5 days before treatment. We routinely tested patients' reactivity to these two antiplatelet drugs and if a patient had a low response to clopidogrel it was replaced with ticagrelor. All interventional operations were performed in our department of interventional neuroradiology by neurointerventionists with more than 10 years of clinical experience. Under general anesthesia, the Seldinger technique was used to puncture bilateral femoral arteries and respectively implant an artery sheath. Two guiding catheters (Codman, Raynham, Massachusetts, USA) were placed into bilateral vertebral arterys for three-dimensional rotatory imaging. Under guidance of a microwire, an Echelon-10 microcatheter was placed into the vertebral artery for coil placement. Marksman (EV3, Irvine, California, USA) was then placed in the P1 segment in the posterior cerebral artery through the contralateral vertebral artery. The PED position was adjusted from multiple angles then carefully released to fully cover the aneurysmal neck. A C-arm flat-detector computed tomography (DynaCT) was then performed to check whether the stent was fully opened and to observe the position of the stent relative to the vessel wall. Flow stagnation inside the aneurysm was assessed to decide whether to insert coils. After the operation, patients were maintained with daily oral 75 mg doses of clopidogrel for 6 months and daily oral 100 mg doses of aspirin for the rest of their life.

### Statistical Analysis

SPSS version 17.0 was used for statistical analysis. Continuous variables were expressed as the mean ± standard deviation and categorical variables were expressed by frequencies. Data comparison between the two groups was conducted by independent sample *t*-test and Fisher's test. Statistical significance was considered at a *p*-value < 0.05.

## Results

### Patient Information and Aneurysm Characteristics

We analyzed 15 males and six females. More male patients were treated with PED alone (*p* = 0.046). Patients ranged in age from 8 to 72 (42.8 ± 20.8) years old. A total of 21 LGVBAs were found in 21 patients: Fourteen were located in the vertebral artery, four in the basilar artery, and three in the junction of the vertebral basilar artery. Of these, 19 were fusiform aneurysms, accounting for more than 90%. It is necessary to mention that seven patients (33.3%) each presented with two aneurysms, but the other aneurysm was not located in the vertebrobasilar artery or was not large (<15 mm). When first admitted into our hospital, all patients were in good condition (mRS = 0–1). Nine patients initially experienced headache, six suffered dizziness, and three patients presented with weakness, choking, and unsteady gait. Aneurysm was an unexpected finding in three patients. Six of the 21 LGVBAs (28.6%) were giant aneurysms. Patients were divided into two groups according to whether PED treatment was or was not combined with coils. There were nine patients in the PED with adjunctive coils group (Table 1) and 12 in the PED group (Table 2). There were no statistical differences in age, smoking, hypertension, aneurysm size, or multiple aneurysm occurrence, between the two groups (Table 3).

**Table 1:** Patient data of the PED with adjunctive coils group.

**Case no**.	**Age(Y) /sex**	**Symptoms**	**Hyp**	**Location**	**LD (mm)**	**No. of PEDs**	**Complications**	**Last angiographic FU (m)**	**Last clinical FU IPS**	**Last clinical FU mRS (m)**
1	34/F	Headache	No	VBJ	18.4	1		D DSA at 14	Resolved	0 at 40
2	8/F	Headache	No	BA	17.6	2		D DSA at 36	Resolved	0 at 36
3	11/F	Headache	No	LVA	25.7	1	RVA occlusion	D DSA at 30	Resolved	0 at 30
4	52/M	Incidentalfinding	Yes	LVA	17.5	1		D CTA at 10	None	0 at 20
5	10/M	Headache choking	No	BA	29.6	2		D DSA at 3	Resolved	0 at 19
6	71/F	Dizziness	Yes	RVA	16.7	1		B CTA at 7	Unresolved	1 at 19
7	39/M	Dizziness	Yes	LVA	16.5	2		C DSA at 4	Resolved	0 at 18
8	56/M	Headache	Yes	LVA	15.3	1	Temporarily Hemiplegia lisp	D DSA at 3	Resolved	0 at 6
9	28/F	Headache	No	VBJ	27.4	1		D DSA at 3	Resolved	0 at 3

*Y, Years old; Hyp, Hypertension; FU, Follow up; IPS, Initial presenting symptoms; m, Months; F, Female; M, Male; BA, Basilar artery; LVA, Left vertebral artery; RVA, Right vertebral artery; VBJ, Vertebrobasilar artery junction; LD, Largest or Longest diameter; BVA, Bilateral vertebral arteries*.

**Table 2:** Patient data of the PED group.

**Case no**.	**Age(Y)/sex**	**Symptoms**	**Hyp**	**Location**	**LD (mm)**	**No. of PEDs**	**Complications**	**Last angiographic FU (m)**	**Last clinical FU IPS**	**Last clinical FU mRS (m)**
10	42/M	Dizziness emesis	Yes	LVA	20.8	1		B DSA at 34	Resolved	0 at 42
11	58/M	Unstable walking	Yes	RVA	20.7	1		D DSA at 10	Unresolved	1 at 40
12	60/M	Incidental finding	Yes	RVA	16.6	1		D DSA at29	None	0 at 39
13	54/F	Dizziness	No	RVA	15.9	1	Hemorrhage	D DSA at 26	Resolved	0 at 36
14	47/M	Headache emesis	No	LVA	20.3	2		D CTA at 34	Resolved	0 at 34
15	61/M	Incidental finding	No	RVA	15.4	1		D DSA at 16	None	0 at 21
16	55/M	Choking	Yes	RVA	21.9	1		B DSA at14	Resolved	0 at 21
17	59/M	Weakness	Yes	RVA	18.8	1		D CTA at 13	Resolved	0 at 17
18	12/M	Headache	No	BA	28.6	4	Died after brainstem infarcation	None	Worsened	6
19	53/M	Dizziness	Yes	RVA	17.2	1		C CTA at 12	Resolved	0 at 16
20	72/M	Dizziness	No	BA	28.8	1	Hemiplegia Dysphagia	C CTA at 3	Unresolved	4 at 3
21	17/M	Headache	No	VBJ	27.5	2	Stent retraction	D CTA at 3	Resolved	0 at 3

**Table 3:** Statistical assessment of parameters of the two groups.

	**Total aneurysms (*N* = 21)** ***n*(%)**	**PED/coil (*N* = 9)** ***n*(%)**	**PED (*N* = 12)** ***n*(%)**	***P*-value**
Age(Y)	42.8 ± 20.8	34.3 ± 22.4	49.2 ± 17.8	0.107
Gender M	15(71.4)	4(44.4)	11(91.7)	0.046
Smoking	5(23.8)	1(11.1)	4(33.3)	0.338
Hypertension	10(47.6)	4(44.4)	6(50.0)	<0.999
LD (mm)	20.8 ± 5.0	20.5 ± 5.4	21.0 ± 4.8	0.848
Operation time	119 ± 44	135 ± 50	107 ± 36	0.155
Complication	6(33.3)	2(28.6)	4(33.3)	0.659
Retreatment	1(4.8)	0(0)	1(8.3)	<0.999
Last angiographic FU/mos (D)	14(66.7)	7(77.8)	7(63.6)*	0.642
Last clinical FU mRS (0–1)	19(90.5)	9(100)	10(83.3)	0.486

*PED/coil, PED adjunctive with coils*.

* *One patient died (case 18); therefore, there was no available data on post-operative angiographic obliteration*.

### Complications

No intraoperative complications occurred in any of the 21 patients. Six patients (28.6%) developed post-operative complications. In the PED with adjunctive coils group, one patient (case 8) had a transient ischemic event. One day after the procedure, the patient manifested unilateral limb weakness, speech difficulties, and other manifestations of focal neurological deficits, but these symptoms quickly disappeared, so we considered the cause to be a vasospasm. In one patient (cases 3) occlusion of the non-treated vertebral artery was observed without any neurological symptoms at the follow-up examination. According to preoperative vertebral arteriography, congenital malformation and existence of coils were considered the main causes of the vertebral artery occlusions. The other four patients were in the PED group: one patient (case 13) developed significant headache and neck pain on the second post-operative day. Cerebral CT showed a small amount of hemorrhage in the lateral fissure pools and cisterna ambiens, indicating that the patient had a ruptured aneurysm. Fortunately, after further observation, the patient became asymptomatic, and follow-up examinations confirmed complete recovery. One patient (case 20) developed post-operative acute intracavitary thrombosis and was treated with intraarterial thrombolysis, the patient recovered to mRS 4. Only one patient (case 18) died. He developed compression of the brainstem and lost autonomous respiration. The last patient (case 21) suffered stent retraction after the operation; therefore, 2 weeks later, he underwent a second interventional embolization to place another PED and he recovered well. All patients, except the patient who died (case 18), underwent post-operative imaging follow-up. The average imaging follow-up time was 15.2 months and ranged from 3 months to 36 months. Fourteen patients achieved complete aneurysm embolization: seven patients (78%) in the PED group and seven (63.6%) in the PED adjunctive with coils group. All patients were followed up clinically for an average of 23.2 months. Nineteen patients (90.5%) achieved favorable outcomes (mRS = 0–1), while case 18 died and the case 20 had an mRS score of 4. There were no significant differences between the two groups for complications, imaging follow-up or clinical follow-up (Table 3).

### Typical Illustrative Cases

**Figure 1:**
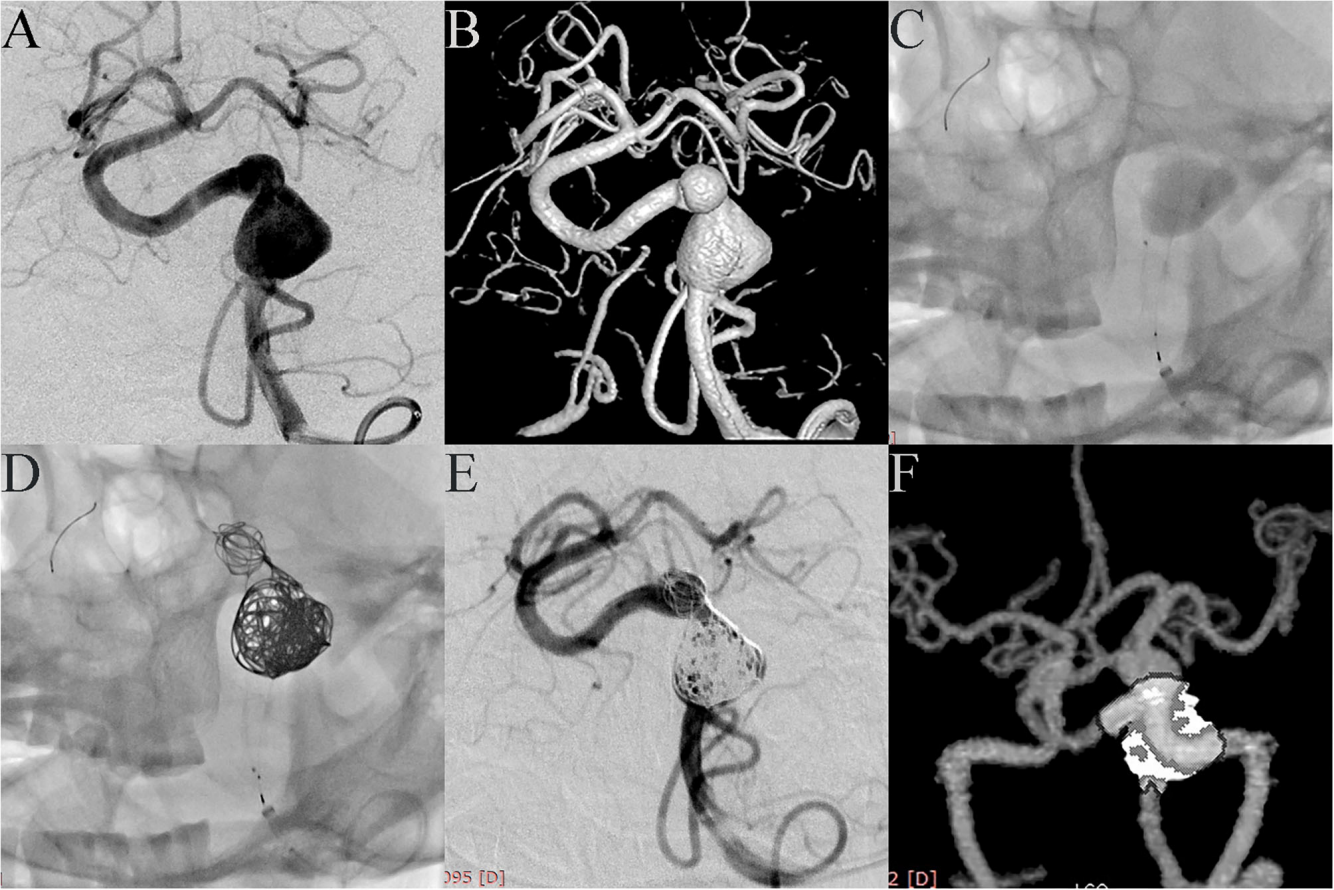
Case 4: Two adjacent fusiform aneurysms were found in the left vertebral artery. (**A**, anteroposterior projection by DSA; **B**, three-dimensional reconstruction). One PED was deployed along the left vertebral artery **(C)**, and both aneurysmal necks were completely covered. Two aneurysm domes were then packed with coils **(D)**. Post-embolization angiograms showed good vascular reconstruction. The large aneurysm was almost completely embolized and the small aneurysm showed significant contrast stasis **(E)**. Follow-up at 10 months with CTA showed complete reconstruction of the vessel with the aneurysm completely occluded **(F)**.

Case 4. A 52-year-old female was admitted with headache. DSA showed two adjacent fusiform aneurysms located in the left vertebral artery (Figures 1A,B). One 4.5 mm × 35 mm Pipeline embolization device was deployed along the left vertebral artery and both aneurysmal necks were completely covered (Figure 1C), then the two aneurysm domes were packed with coils (Figure 1D). Post-embolization angiograms showed good vascular reconstruction; the large aneurysm was almost completely embolized and the small aneurysm showed significant contrast stasis (Figure 1E). Follow up at 10 months by CTA showed complete reconstruction of the vessel with complete occlusion of the aneurysm (Figure 1F).

**Figure 2:**
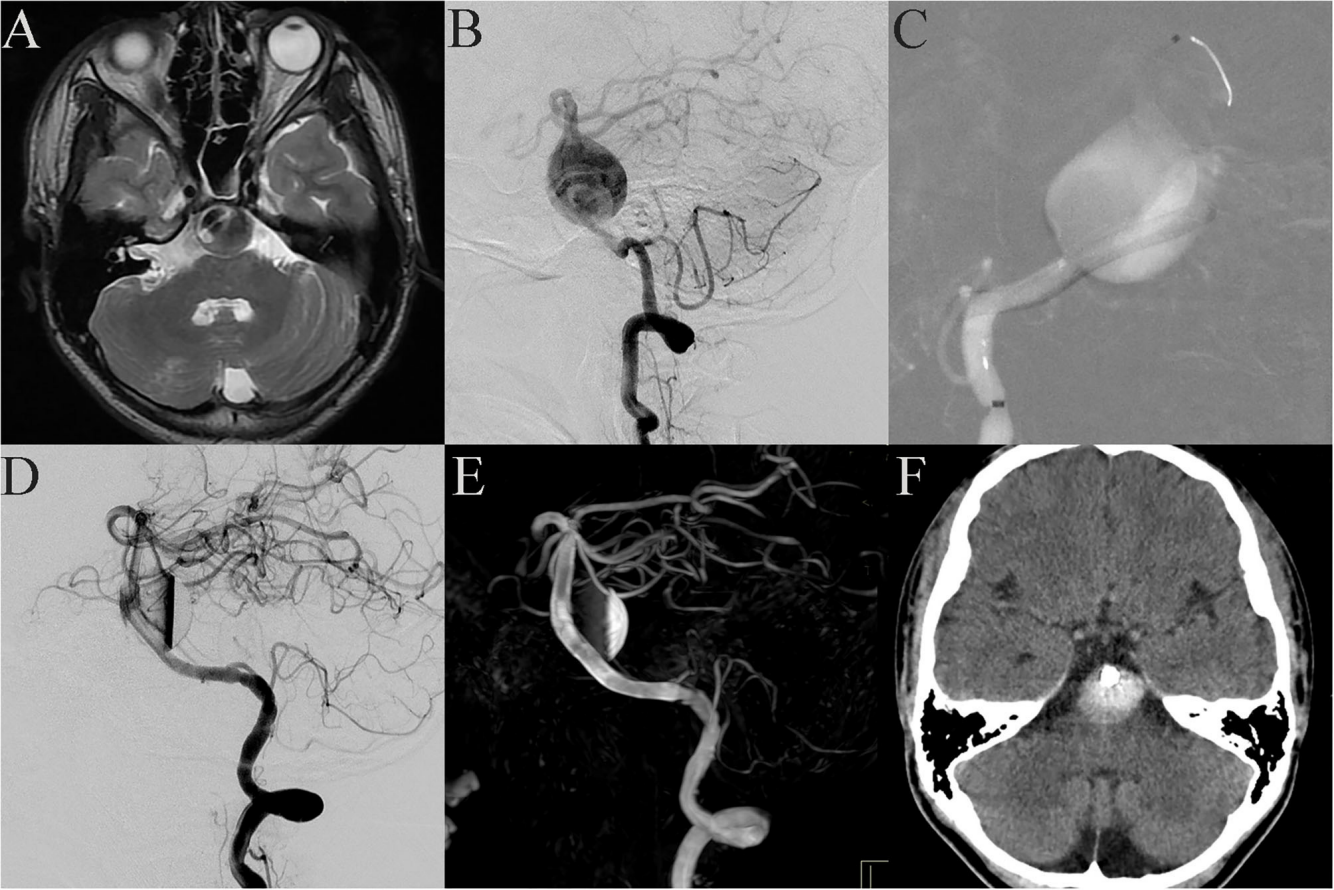
Case 18: Basilar trunk containing a giant fusiform aneurysm (**A**, MRI; **B**, lateral projection by DSA). During the stent bridging, the distal end of the stent easily fell into the aneurysm cavity **(C)**. Post-embolization angiograms showed contrast stasis **(D)**. Dyna CT showed good vascular reconstruction **(E)**. One day post-operation, the patient gradually presented dyspnea and hemiplegia. Emergent cerebral CT showed no obvious bleeding **(F)**.

Case 18: A 12-year-old patient was admitted to the hospital with a headache as the main symptom. MRI showed that a giant aneurysm compressed the brain stem (Figure 2A), and DSA showed a giant fusiform aneurysm in the basilar trunk (Figure 2B). The patient received PED treatment. Because of its large size, the vessel wall of the aneurysm was not long enough to support the stent and the neurointerventionist had to use multiple PEDs bridging technology. The process was difficult because the distal end of the PED easily fell into the aneurysm cavity (Figure 2C). A fourth PED was therefore used to anchor the distal end of the stent to the posterior cerebral artery. Post-embolization angiograms showed contrast stasis (Figure 2D). Dyna CT showed good vascular reconstruction (Figure 2E). However, 1 day post-operation, the patient gradually presented dyspnea and hemiplegia. Emergent cerebral CT showed no obvious bleeding (Figure 2F), and symptomatic treatment relieved these symptoms. However, 3 days post-operation the patient gradually lost consciousness and experienced spontaneous apnea. Because the brain stem was compressed, the patient was immediately sent to the intensive care unit, where, despite active mechanical ventilation, the patient died.

**Figure 3:**
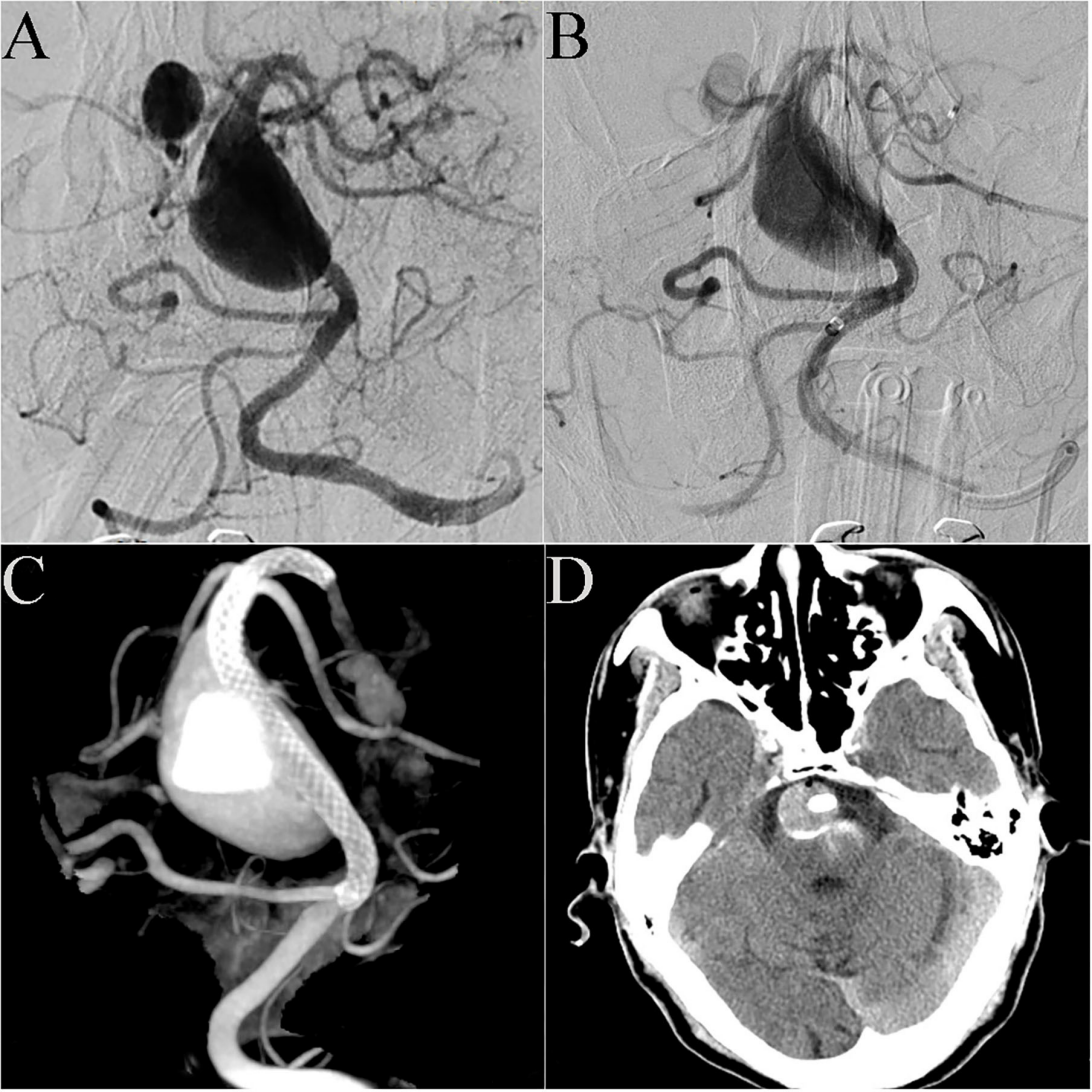
Case 20: DSA showed that the patient had a giant dissecting aneurysm of the basilar artery and a posterior cerebral arterial aneurysm **(A)**. A 4.5 mm × 35 mm PED was successfully deployed in the basilar artery. Immediate post-embolization angiograms showed contrast stasis in the lumen of the aneurysm **(B)**. Dyna CT showed good vascular reconstruction **(C)**. Routine CT scanning showed no obvious bleeding or infarction on post-operative day 2 **(D)**.

Case 20: A 72-year-old man presented with sudden dizziness, nausea, and vomiting. DSA showed that the patient had a basilar artery giant dissecting aneurysm and a posterior cerebral artery aneurysm (Figure 3A). Preoperative discussion led to the decision to address the basilar artery aneurysm in a first operation. A 4.5 mm × 35 mm PED was successfully placed in the basilar artery. Immediate post-embolization angiograms revealed the aneurysm neck was completely covered by the PED and significant contrast stasis (Figure 3B). Dyna CT showed good vascular reconstruction (Figure 3C). Routine CT scanning showed no obvious bleeding or infarction on the post-operative day 2 (Figure 3D), and the patient was discharged. However, 1 week post-discharge, the patient gradually showed obvious hemiplegia, dysphagia and difficulty speaking. The patient was admitted to the local hospital. Cerebral CT (via remote consultation) showed no bleeding. Because intracavitary thrombosis compressed the brain stem, the patient was administered low-molecular weight heparin, which partially relieved his symptoms. At the 3-month follow-up, he presented with dysphagia and unsteady gait; his muscle strength had improved to level 4, and his mRS score had improved to 4.

**Figure 4:**
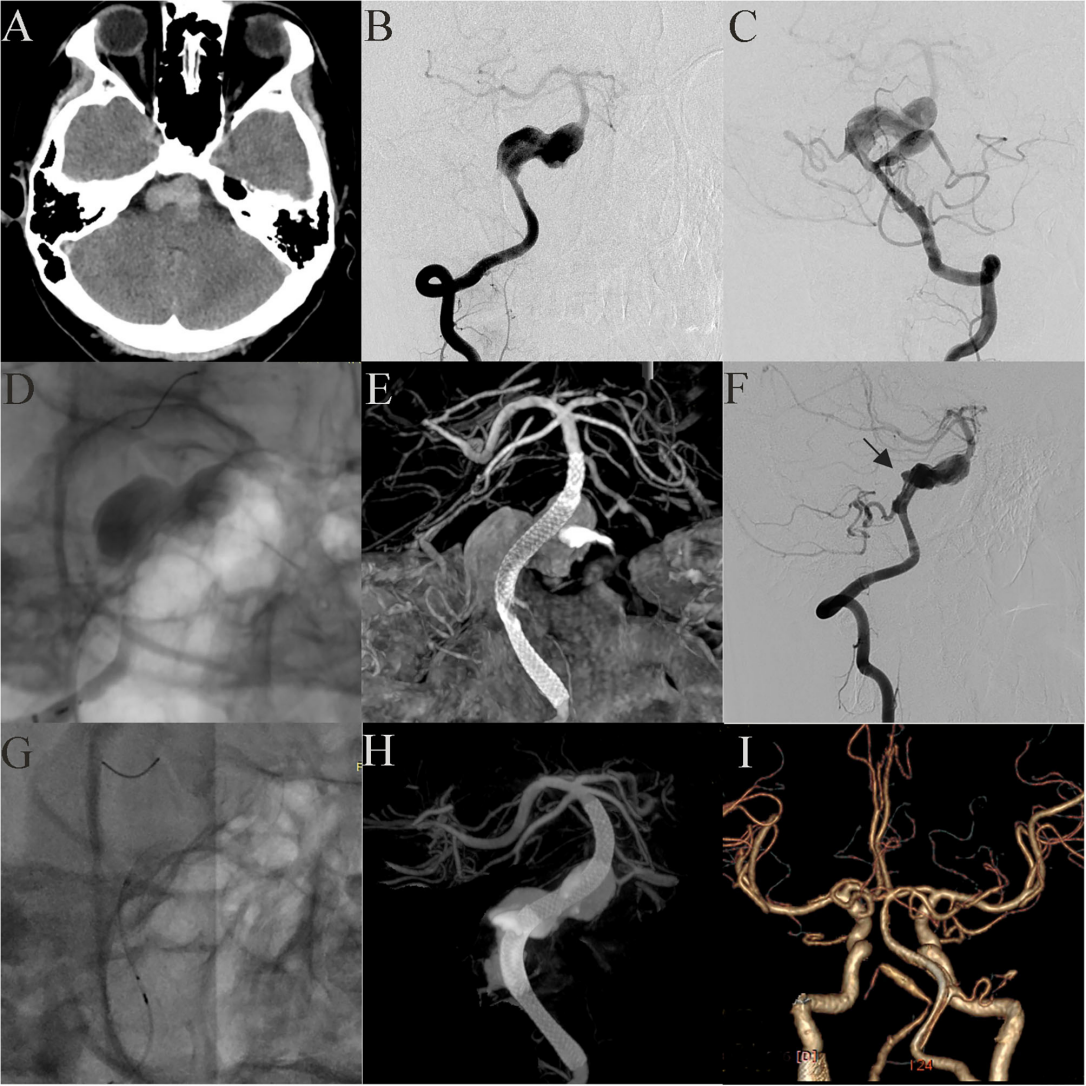
Case 21: A 17-year-old boy had a giant vertebrobasilar artery aneurysm and a PICA aneurysm (**A**, CT scanning; **B, C**, anteroposterior projections of bilateral vertebral arteries). A 3.75 mm × 35 mm PED was successfully deployed from the basilar artery to the right vertebral artery. Post-embolization angiograms showed significant contrast stasis **(D)** and good vascular reconstruction **(E)**. The left PICA aneurysm was untreated. Two weeks later, the distal end of the stent (black arrow) retracted and fell into the aneurysm cavity **(F)**. Another PED was used to bridge the original PED **(G, H)**. Three months later, CTA examination showed that the aneurysm was completely occluded **(I)**.

Case 21: A 17-year-old boy presented with headache and extremity numbness and had a giant dissecting fusiform vertebrobasilar artery aneurysm and a posterior inferior cerebellar artery (PICA) aneurysm (Figures 4A–C). The preoperative plan determined that two operations should be performed to individually treat the vertebral aneurysm and the PICA aneurysm. To treat the vertebral aneurysm: a 3.75 mm × 35 mm PED was successfully deployed along the basilar artery and right vertebral artery. A post-embolization control angiogram showed significant contrast stasis (Figure 4D) and Dyna CT showed good vascular reconstruction (Figure 4E). The left PICA aneurysm was untreated. Two weeks later the patient felt a headache again and DSA showed that the distal end of the stent (Figure 4F, Black arrow) had retracted and fallen into the aneurysm cavity. A second operation was performed where another PED was bridged with the previous PED (Figure 4G). A post-embolization control angiogram showed significant contrast stasis and Dyna CT showed good stent adherence and vascular reconstruction (Figure 4H). Three months later, follow-up CTA examination demonstrated that the aneurysm was completely occluded (Figure 4I) and the patient showed no nervous system symptoms. The patient still needs an operation to manage the PICA aneurysm and long-term follow-up will be necessary.

## Discussion

Intracranial large and giant aneurysms of the posterior circulation are difficult to treat surgically, especially when in the basilar trunk (15). Unlike most anterior circulation saccular aneurysms, the former tend to present as irregular fusiform or dissecting aneurysms. In our study, fusiform aneurysms accounted for 90.5%. Nasr et al. (16) analyzed 827 patients with vertebrobasilar non-saccular and dolichoectatic aneurysms (VBDAs) from fifteen studies. The natural history of VBDAs was bad; the overall annual mortality rate among patients was 13%/year (95% CI 8–19). Since the discovery of LGVBAs, neurosurgeons have tried various methods to treat them. Because of their particular anatomical characteristics, being adjacent to the brainstem and containing many important branch vessels, surgical treatments, such as surgical bypass, and clip reconstruction techniques, have high rates of recurrence and complications (17, 18). Kalani et al. (19) reported 11 patients with 12 posterior circulation giant aneurysms. All patients were treated with bypass and at last follow-up for all patients. Five patients died, and the other six patients' average mRS score was 2.5. Interventional embolization has become the technique of choice for the treatment of LGVBAs (5); however, early intervention methods were not effective because of the large aneurysm cavity and loss of effective vascular wall inhibiting stent-assisted coil embolization and vascular remodeling. Wu et al. (11) reported nine patients with symptomatic vertebrobasilar dolichoectasias treated with stent-assisted coiling. The result was poor: two patients had brainstem infarct and two patients died. In addition, the method of artery occlusion carries a risk of immediate or delayed ischemia and is only applicable for patients who have better compensatory posterior communicating arteries (20). The emergence of flow diverters provides a possibility to overcome these problems and they have become the first choice for LGVBAs treatment.

PED, a type of flow diverter, was originally designed for aneurysms in the internal carotid artery. However, with the accumulation of treatment experience, the off-label use of PED has become common. Neurointerventionists have tried to use it for refractory aneurysms in posterior circulation, although initial results were not ideal. Siddiqui et al. (8) reported seven LGVBA patients treated with PED. Four patients died and only two patients achieved favorable outcomes at long-term follow-up. Similarly, Toth et al. (9) reported seven patients treated with PED for posterior circulation aneurysm. Three patients with LGVBAs were treated with PED embolization, two of which had serious neurological symptoms. Toth et al. blamed the unsatisfactory results on most patients having obvious neurological symptoms when admitted to hospital and all patients receiving multiple PEDs bridging technology. Learning from these lessons, Natarajan et al. (6) reported 12 posterior circulation aneurysms treated with PED. Eleven patients achieved good clinical results. Five of these LGVBA patients received coil-assisted embolization and all had favorable outcomes. Our results here are similar; 19 (90%) of the 21 LGVBA patients achieved favorable outcomes, nine patients in the PED with adjunctive coils group (100%) all had favorable outcomes, 10 patients in PED group (83%) had favorable outcomes. Although there was no statistical difference between the two groups in clinical and imaging follow-up, our results indicated that PED combined with coils did not increase perioperative or long-term complications.

### Complication

Branch vessels of intracranial posterior circulation are abundant and important. Once occluded, they pose a risk of immediate or delayed ischemia, for areas supplied by the PICA, anterior inferior cerebella artery (AICA) and basilar perforating arteries. Many studies have attempted to show the influence on these branches after PED implantation. Lall et al. (10) described three patients who were each treated by placement of a single PED. Shortly after placement of the device, despite adequate antiplatelet and anticoagulation regimens, partial or complete occlusion of a major side branch occurred. In all three patients, the occlusion was promptly reversed with intra-arterial administration of abciximab with no clinical sequelae. Wu et al. (21) studied 18 cases with patency of posterior circulation branches covered by a flow diverter device. Computational fluid dynamics results showed that a flow diverter does not slow down the flow velocity of covered branches. Daou et al. (22) reported 30 patients with posterior communicating artery (PCoA) aneurysms who were all treated with PED. At a six-month follow up, the PCoA was patent in only seven patients, and no patients had symptoms related to PCoA occlusion. In our study, one patient developed occlusion of the non-dominant vertebral artery without associated neurological symptoms. It seems that PED has a high risk of branch vessel occlusion. However, few patients developed clinically related neurological symptoms. Despite this, it is wise to avoid covering important branch vessels when deciding where to place the device. Multiple bridge technology is likely to increase the risk of branch infarction. The metal surface area coverage of a PED is over 30% and would be much higher with stent overlapping, which limits blood flow causing branch infarction. In Siddiqui et al.'s study, patients all received multiple stents (an average of 5.3 PEDs per patient). Similarly, the only patient that died in our study (case 18) received the largest number of PEDs. But beyond that his giant aneurysm was located in the middle of the basilar artery trunk, which is adjacent to the brainstem and has abundant branches. We therefore thought these factors contributed to the cause of death. We believe that although larger aneurysms usually require a higher number of PEDs, the number of devices should be minimized under the premise of finishing vascular reconstruction, which may be achieved through auxiliary coils. In addition, intrastent thrombosis after stent placement is an important reason of infraction. Thus in our institution, dual antiplatelet agents are strictly administered after surgery: clopidogrel 75 mg/day for at least six months and aspirin 100 mg/day for life. Aneurysm rupture after PED placement is also a concern as confirmed in some reports. Fox et al. (7) reported a case of early aneurysm rupture and Rehman et al. (23) reported a case of early parental artery rupture after PED placement. Both patients died and underwent autopsy, which ruled out iatrogenic etiology, such as wire perforation or vessel dissection; they thought the PED placement may have produced radial mechanical tension, which may have led to aneurysm or parental artery rupture. In addition, treatment with flow devices changes intra-aneurysmal hemodynamics, which can increase the risk of early aneurysm rupture (24). Subsequent factors may contribute to aneurysm rupture, such as thrombus formation and inflammatory response (25), but these factors have yet to be confirmed.

### PED With Adjunctive Coils

Currently, it is controversial to combine PED with coils to treat intracranial aneurysms. The choice of this method is entirely based on the individual judgement of the neurointerventionist. Combined PED and coil embolization can speed up the complete embolization of aneurysms and reduce the recurrence rate of aneurysms (12, 26). At the same time, it also increases the complexity of the operation, while there is no consensus on whether it increases the overall complication of the operation (13). The following is our experience on using combined PED and coil embolization. First, we think that PED with adjunctive coils embolization can accelerates thrombus formation in the aneurysmal sac, which can help the aneurysm heal and prevent it from rupturing further. This is suitable for aneurysms that are prone to rupture such as irregular aneurysms with accessory ascus or aneurysms that show visible growth on imaging (27, 28). Second, existence of the coil can reduce the risk of the PED shortening or migrating. Stent shortening may lead to aneurysm recanalization and, more severely, allow blood to be injected directly into the aneurysm wall leading to aneurysm rupture (29). Two patients in the PED group (cases 18 and 21) showed stent shortening during or after the operation, thus requiring additional PEDs, which increased the riskiness of the procedure and of subsequent complications. It also increased the treatment cost. Therefore, it is necessary to accurately measure the diameter of the parental artery and to select a device of appropriate diameter and length. If there is risk of stent movement, especially with giant aneurysms, we recommend the coil combination. Third, PED with adjunctive coils can effectively treat ruptured saccular aneurysms. Brinjikji et al. (30) studied 27 patients diagnosed with ruptured complex large or giant aneurysms. Four aneurysms were in the basilar artery, and one was in the PICA. All patients underwent standard endovascular coiling in the acute phase after SAH and proceeded with flow diversion at a later date (mean: 16 weeks). At the last follow-up, five patients had mRS scores ≤2. However, intraoperative coil placement affect stent visibility, and coils are a risk factor for thrombotic events and increase the mass effect. Oel et al. (14) reported 13 basilar fusiform aneurysm patients treated with laser-cut stents and coils. Three patients had subarachnoid hemorrhage and two of them died. Therefore, in our institution, we usually select 1–2 long type coils for loose packing of the aneurysm cavity considering dense packing may increase the risk of aneurysm rupture. It is also important to emphasize that when aneurysms involve major branch vessels, we oppose the use of combined coils. Compared with the anterior circulation aneurysms, large aneurysms in the posterior circulation are more complex. No study had previously addressed how PED combined with coils performs in this area; therefore, we present our research of 21 patients.

## Limitation

This is a single-center retrospective study. Given the small number of patients with large and giant aneurysms in the posterior circulation who were treated with PED, the study did not have enough power to detect a statistically significant difference between treatments. In this study, some patients lacked long-term radiographic follow-up data. This study is limited by the inclusion of pediatric patients who differ pathophysiologically from adults and who required longer follow-up time. In addition, we did not check the data of the patients' response to aspirin and clopidogrel, or strictly distinguish the types of aneurysm. Further multicenter research is required to provide stronger evidence to support these preliminary results.

## Conclusion

The effectiveness and safety of PED for LGVBAs are acceptable. Treatment results did not differ between the PED and PED with adjunctive coils groups; therefore, whether coils should be used may depend the operator. Our results suggest that correct use of the coils does not increase complications. We suggest that PED with adjunctive coils should be used for selected LGVBAs, although continued exploration of the application is warranted.

## Data Availability Statement

The datasets generated for this study are available on request to the corresponding author.

## Ethics Statement

The studies involving human participants were reviewed and approved by ethics board of Beijing Tiantan Hospital. Written informed consent to participate in this study was provided by the participants' legal guardian/next of kin.

## Author Contributions

YZ collected the clinical data, performed the statistical analysis, and wrote the manuscript. XW and ZT helped collect the clinical data. XW, XY, and SM helped revise the manuscript. SM designed the research and handled funding and supervision. All authors read and approved the final manuscript.

## Conflict of Interest

The authors declare that the research was conducted in the absence of any commercial or financial relationships that could be construed as a potential conflict of interest.
